# Winds of Change in Antibiotic Therapy following Orthognathic Surgery

**DOI:** 10.3390/medicina58020263

**Published:** 2022-02-10

**Authors:** Oren Peleg, Reema Mahmoud, Clariel Ianculovici, Shimrit Arbel, Shlomi Kleinman, Amir Shuster

**Affiliations:** 1 Department of Otolaryngology Head and Neck Surgery and Maxillofacial Surgery, Tel-Aviv Sourasky Medical Center, Sackler School of Medicine, Tel Aviv University, Tel Aviv 642 3906, Israel; orenp@tlvmc.gov.il (O.P.); clarielc@tlvmc.gov.il (C.I.); shimrita@tlvmc.gov.il (S.A.); shlomik@tlvmc.gov.il (S.K.); amirshu@tlvmc.gov.il (A.S.); 2 Maurice and Gabriela Goldschleger School of Dental Medicine, Tel Aviv University, Tel Aviv 642 3906, Israel

**Keywords:** orthognathic surgery, antibiotic prophylaxis, surgical-wound infection, postoperative complications

## Abstract

*Background and objectives*: This retrospective cohort study aimed to compare three postoperative antibiotic protocols of different durations on surgical-site-infection (SSI) rates following orthognathic surgery for the correction of jaw deformities. *Materials and methods*: An analysis on data collected from the medical files of 209 patients who underwent orthognathic surgery between 2010 and 2019 was conducted. The patients were divided into three groups according to the postoperative antibiotic protocol—Group 1 (24 h), Group 2 (2–3 days), and Group 3 (>3 days). Dependent and independent variables were collected, analyzed, and compared between the three groups. *Results*: Group 1 included 30 patients (14.3%), Group 2 included 123 patients (58.9%), and Group 3 included 56 patients (26.8%). The vast majority of the postoperative antibiotics were amoxicillinand clavulanic acid (87.1%). The duration of the surgery and the use of a feeding tube were significantly different between Groups 1 and 3 (*p* < 0.001 and *p* = 0.005, respectively). There was no significant difference in SSI rates between the three groups (*p* = 0.642). The use of antibiotics beyond the immediate postoperative period provides no increased benefit regarding infection prevention. *Conclusions*: In young and healthy patients undergoing orthognathic surgery, a 24hregimen of postoperative antibiotics may be sufficient.

## 1. Introduction

Jaw deformities can occur in childhood and adulthood, and even during adult life, and they can adversely affect aesthetics, function, and a patient’s quality of life. Orthognathic surgery aims to correct these deformities, which cannot be corrected with orthodontic treatment alone, in order to normalize the relations between the jaws, skull, and surrounding facial tissues. The most common surgical procedures are surgically assisted rapid palatal/maxillary expansion (SARPE/SARME), Le Fort I osteotomy, sagittal split osteotomy (SSO), intraoral vertical ramus osteotomy (IVRO), and genioplasty. These surgical procedures are performed in the microbial-rich environment of the oral cavity, nasal cavity, and maxillary sinuses, with postoperative infections occurring in 1.4% to 33.4% of cases [1,2,3].

Chow et al. discussed the need for postoperative antibiotics to lower the rate of surgical-site infections in orthognathic surgery [4]. A meta-analysis by Danda suggested that extended postoperative antibiotic therapy plays a role in decreasing the surgical-site infection risk in orthognathic surgery [3]. Similar findings presented by Davis showed a decreased postoperative infection rate when a postoperative antibiotic protocol of 3 days was followed rather than a single-day protocol [5].

Conversely, Kang found no significant difference in the incidence of postoperative wound infections between patients receiving a postoperative 1.0 g of cefpiramide twice daily for 3 days and those who did not receive any antibiotic therapy [6]. Lindeboom compared the incidence of postoperative infections following orthognathic surgery among one group of patients who received a single prophylactic dose of clindamycin postoperatively and another group that received a 24h clindamycin regimen, and found no significant difference, suggesting that the use of postoperative clindamycin is unnecessary [7].

Postoperative infections may require additional hospital care and, sometimes, even further surgical interventions, creating a burden on the medical system and discomfort to the patient [8]. The common use of antibiotics is, however, far from being harmless. Apart from minor adverse events, such as nausea, diarrhea, and vomiting, the overuse of antibiotics contributes directly to the increasing microbial drug resistance [9].

To date, there is no consensus regarding the optimal antibiotic protocol for orthognathic surgery [3,4,5,6,7]. Therefore, the purpose of the present study was to compare the consequences of a 1-day regimen, a short-term regimen (2–3 days), and an extended-term regimen (>3 days) of postoperative antibiotics forthe surgical site infection rate in the setting of orthognathic surgery for correcting deformities of the jaw.

## 2. Materials and Methods

### 2.1. Study Design and Ethics

This retrospective cohort study was conducted according to the “Strengthening the Reporting of Observational Studies in Epidemiology” (STROBE) checklist [10] based on data collected from the medical files of patients who had undergone an orthognathic surgery in the Tel-Aviv Sourasky Medical Center (TASMC), Israel, between January 2010 and December 2019. During this period, five senior surgeons participated in orthognathic surgeries.

Approval was obtained from the institutional Helsinki Ethics Committee on 21 June 2020 (0300-20-TLV), which waived the requirement for informed consent. Patients of all ages were identified according to the procedure codes of The International Classification of Diseases, ninth revision (ICD9) [11].

### 2.2. Patient Selection

All of the patients who had undergone an orthognathic surgery involving SARPE/Le Fort I/SSO/IVRO were identified. Patients were excluded if the surgical procedures with the same ICD9 codes were used for purposes other than orthognathic surgery or if documented information regarding the surgical procedure (IVRO/SSO/LEFORT/SARPE) or regarding the postoperative antibiotic use waslacking.

The patients were divided into three groups according to the postoperative treatment protocol that consisted of any antibiotic type. Group 1 included patients who received 1 day of postoperative antibiotics, Group 2 included patients treated with 2–3 days of postoperative antibiotics, and Group 3 included patients treated with a prolonged antibiotic treatment (>3 days). The patients were evaluated daily during their hospital stays. A clinical and radiographic evaluation was carried out during outpatient follow-up visits.

### 2.3. Data Collection

Dependent and independent variables were collected from the entire cohort’s medical files and entered into structured Excel sheets. The independent variables included demographics (age and sex);details of the surgical procedure (type, duration, and whether another procedure had been performed concomitantly);the type, dosage, and route of the administration of prophylactic and postoperative antibiotics and steroids;the length of hospitalization; the use of a urinary catheter and feeding tube;and the follow-up duration. Surgical-site infection (SSI) was considered a dependent variable. SSI was diagnosed according to the definition of the CDC/NHSN (Centers for Disease Control and Prevention/National Healthcare Safety Network) surveillance criteria [12], which states that infection must have been diagnosed within 30 days after the operative procedure, that it involves only skin and subcutaneous tissue of the incision, and that the patient must have had at least one of the following:

1. Purulent drainage from the superficial incision.
2. An organism isolated from a specimen obtained aseptically from the superficial incision or subcutaneous tissue.
3. A superficial incision that was deliberately opened by a physician conducting culture- or non-culture-based testing of the superficial incision or of the subcutaneous tissue, provided that the patient hadat least one of the following signs or symptoms: localized pain or tenderness, localized swelling, erythema, or warmth.
4. A diagnosis of a superficial incisional SSI by a physician.

For each patient identified as having a postoperative SSI, detailed information was collected regarding the onset, duration, relevant blood test results (complete blood count and a chemistry panel blood test), management, and outcome.

### 2.4. StatisticalMethods

Categorical variables are reported as numbers and percentages. The Shapiro–Wilk test was used to evaluate the normal distributions of continuous variables. Continuous variables are presented as medians and interquartile ranges (IQRs). The Kruskal–Wallis test and the Mann–Whitney test were used to compare continuous variables between the three antibiotic protocols. Fisher’s exact test and the chi-squared test were applied to compare categorical variables. All the statistical tests were 2-sided. A *p*-value <0.05 was considered statistically significant. The NCSS 2020 software was used for all the statistical analyses (“NCSS 2020 Statistical Software (2020). NCSS, LLC. Kaysville, UT, USA”).

## 3. Results

### 3.1. Participants

A total of 473 patients were enrolled in the study from January 2010 through December 2019, of whom 138 were excluded due to duplications, 98 had surgical procedures other than orthognathic surgery, and 28 did not have detailed information on the postoperative antibiotic use, leaving 209 patients eligible for study entry.

For mandible procedures, IVRO was used for mandibular setback, while SSO was used only for mandibular advancement. During bimaxillary procedures, the maxilla was operated on first.

### 3.2. Descriptive Data

The study group of 209 patients consisted of 77 males (36.8%) and 132 females (63.2%) with a median age of 21 years (IQR: 18–25 years). Sixty-seven of the 79 patients (37.8%) who had undergone a single-jaw surgical procedure had maxillary procedures (32.1%), while the remaining 12 patients had mandibular procedures (5.7%). The single-jaw procedures were divided as follows: 37 SARPEs (17.7%), 30 LeFort I osteotomies (14.3%), twoIVROs (1%), and 10 SSOs (4.8%). The remaining 130 patients (62.2%) underwent surgery involving both jaws: 115 patients (55%) had an IVRO+LeFort I procedure, while 15 patients (7.2%) had an SSO+LeFort I procedure. Thirty patients (14.3%) had an additional osteotomy involving the chin (genioplasty).

The median duration of surgery was 4.025 h (IQR: 2.96–4.67), the median number of hospitalization days was 6 (IQR: 5-7), and the median follow-up period (weeks) was 7.86 (IQR: 3.43–28.29). The overall SSI rate was 3.3% (sevenpatients).

### 3.3. Results According to Group Allocation

The patients were assigned to one of three groups according to the postoperative antibiotic protocol they followed: Group 1 (1 day), Group 2 (2–3 days), and Group 3 (>3 days). The groups included 30 (14.3%), 123 (58.9%), and 56 (26.8%) patients, respectively. The vast majority received an intravenous postoperative protocol of 1g of amoxicillin–clavulanic acid three times a day (87.1%), while the penicillin-sensitive patients received 600mg of clindamycin three times a day (12.9%). All the patients received steroids preoperatively (dexamethasone,20 mg, Rekah, Israel). The characteristics of each group are listed in Table 1.

All three groups were statistically comparable with regard to the male–female ratio, age, surgical procedure, catheter use, preoperative antibiotic use, hospitalization days, postoperative steroid use, and follow-up period. However, the duration of the surgery and the use of a feeding tube were significantly different (*p* = 0.019 and *p* = 0.008, respectively).

Specifically, the differences in the use of a feeding tube and the duration of surgery between Groups 1 and 3 were *p* < 0.001 and *p* = 0.005, respectively. There was a significant difference between Groups 1 and 2 only in the use of a feeding tube (*p* = 0.001), while there was no comparably significant difference between Groups 2 and 3.

### 3.4. SSI According to Group Allocation

There was no significant difference in SSI rates between the three groups, with one event of SSI in Group 1 (3.3%), and three events of SSI each in Group 2 (2.4%) and Group 3 (5.4%) (*p* = 0.642) (Table 2). Two of the three patients with SSI in Group 2 and all three patients in Group 3 required the draining of an abscess under general anesthesia. The patient with an SSI in Group 1 did not require any further surgical treatment, yet he was hospitalized and treated with amoxicillin–clavulanic acid for a week. There was no significant difference in the severity of infection between the three groups (*p* = 0.57). We further studied the relationship between numerous variables and SSI utilizing a cross-tabulation method, and the results show that males (*n* = 6) were significantly more likely to sustain an infection (*p* = 0.01) (Table 3).

No significant relation between the SSI rates and the osteotomy type, catheter and feeding-tube use, preoperative antibiotic use, and postoperative steroid usewas observed.

In addition, general culture and direct smear tests revealed three cases with a mixed growth of Gram-negative rods and Gram-positive cocci in our study population; all of them were in the third group: Streptococcus viridans was isolated in two cases, while Enterobacter cloacae was identified in the third patient’s culture.

Table 4 lists the median and IQR values of the continuous variables, including the age, operation duration, hospitalization, and follow-up periods of those who sustained an SSI and those who were infection-free, again revealing no significant differences between them.

## 4. Discussion

Jaw deformities can develop at any phase of life and can adversely affect the aesthetic and functional aspects of the patient’s quality of life. Orthognathic surgery aims to correct these deformities. The surgical procedures are performed in the microbe-rich environment of the oral cavity, nasal cavity, and maxillary sinuses, and are classified as clean-contaminated procedures. Their acceptance is worldwide, and there has been a marked increase in their application for correcting congenital and acquired dentofacial discrepancies [13]. The use of antibiotic prophylaxis reduces the incidence of infections in these procedures [7], but the duration of postoperative antibiotic protocols differs, and the issue remains controversial [3,5,14,15].

SSI continues to be the second most common cause of healthcare-associated infections in Europe and the USA [16].Due to the anxiety associated with postoperative SSI, many surgeons tend to prescribe antibiotics without considering their adverse side effects. This overuse leads to a rise in antibiotic-related adverse events and an increase in microbial drug resistance [9,17]. Moreover, it has been estimated that about one-half of SSI cases can be prevented by using evidence-based protocols [18]. For example, in maxillofacial trauma, a short-term antibiotic therapy, e.g., a single dose or a 24h protocol, appears to be effective in lowering SSI rates in compound mandibular fractures [19].

The goal of the current work was to test whether a short, single-day postoperative antibiotic protocol wouldincrease the incidence of infections after surgery. We hypothesized that the occurrence of SSI would remain low, with results comparable to those of other postoperative antibiotic protocols of longer duration. Our study findings revealed a low infection rate (3.3%) compared to the rates reported in the literature [3,6].

There was no significant difference in infection rates between our patients who received single-day, 2–3-day, or >3-day postoperative antibiotic protocols. Additionally, the severity of the infection, as reflected by the need to drain the abscess under general anesthesia, was not significantly different between the three study groups. The vast majority of the selected postoperative antibiotic protocolswereamoxicillin–clavulanic acid based upon its antimicrobial activity against typical oral bacteria [20]. Bentley et al. compared a single-day regimen to a 5-day regimen of antibiotics after orthognathic surgery and found infection rates of 60% and 6.7%, respectively [14]. Our study results were comparable to those of Ghantous, who claimed that prolonged antibiotic treatment after orthognathic surgery didnot necessarily reduce the incidence of SSI [9].Those authors found no significant differences in the mean postoperative C-reactive-protein levels, body temperature, and infection rates between two groups of young, healthy patients, among whom one group received 1 g of intravenous amoxicillin–clavulanate three times a day for 5 days, and the second group received a placebo for the same period of time.

Since our study was retrospective in design, which precluded the randomization of the patient assignment, we were obliged to examine other possible factors that could theoretically have influenced the decision of whether or not to administer prolonged antibiotic therapy.

Processing the statistical methods forthe variables of age, the use of preoperative steroids and antibiotics, the use of a urinary catheter, and the osteotomy type did not reveal any significant difference between the three study groups. The operation duration and the use of a feeding tube, however, did differ significantly between them. Posnick stated that the combination of a meticulous surgical technique, limited open-wound-operating time, and administration of a prophylactic antibiotic should decrease infection rates [21].

In the absence of a binding protocol, the duration of antibiotic treatment was mainly determined by the perception of the senior physician who operated on the patient. Interestingly, only one patient suffered from an infection while being hospitalized thatrequired prolonging the antibiotic treatment.

Furthermore, the lack of significant difference between the groups in terms of the length of hospitalization may indicate that the protracted stay was not necessarily related to the antibiotic protocol, but to what is customary in our hospital in these kinds of surgeries.

It should be noted that, according to our department’s protocol, all patients who undergo orthogenetic surgery receive prophylactic antibiotics (a preoperative 1g of intravenous amoxicillin–clavulanate, or 600mg of clindamycin when the patient is sensitive to penicillin is administered before the first incision is made). Therefore, regarding the four patients about whom it is not stated whether they received a preoperative dose or not, we assume that they actually did receive it but, for some reason, it was not recorded.

Similar to that in other studies, the SSI among our study patients occurred mainly in the mandible (85.7%). Mandibular SSOs and IVROs are more susceptible to SSIs than maxillary Le Fort I osteotomy and SARPE procedures, since the mandible is less vascular than the maxilla and more exposed to saliva and food debris that can contaminate the incisions in the mandibular vestibule during the soft-tissue-healing process [3,5].

We postulate that the use of antibiotics beyond the immediate postoperative period provides no increased benefit regarding infection prevention. This assumption is based on the lack of any significant difference among our three study groups regarding the relationship between the antibiotic treatment protocol and SSI incidence. Not only were the incidences of SSI considered low in all the study groups, but also, continuing the antibiotic treatment beyond 3 days did not lower the complication rate. In addition, Chow et al. observed that following an extended-course regimen meantextending the hospital stay, increased treatment costs, excessive fatigue of the medical staff, and a hassle to the patient and their family [4]. It had also been reported that extended-course participants had significantly higher treatment costs per participant than short-course participants [22]. We anticipate that there may be a downward trend in the length of the hospitalization of patients undergoing orthognathic surgery due to the decrease in the duration of antibiotic therapy.

## 5. Conclusions

Our work supports the mind shift towards the administration of a single-day postoperative antibiotic protocol in young, healthy patients undergoing orthognathic surgery, in order to decrease microbial drug resistance, hospital stays, and overall treatment costs.

## Figures and Tables

**Table 1:** Characteristics of the study groups.

Characteristic	Group 1	Group 2	Group 3	*p*-Value
Age (years)				0.322
Median	23	21	20	
IQR	18.75–26.25	18–25	18–24	
Operation Duration (hours)				0.019
Median	3.63	4	4.25	
IQR	1.63–4.5	2.93–4.68	3.79–4.83	
Hospitalization (days)				0.626
Median	7	6	6	
IQR	4.75–7	4–7	5–7	
Follow-up (weeks)				0.681
Median	8.14	8.07	6.71	
IQR	3.86–16.86	3.29–39.86	3.57–25.14	

*Group 1*: one day of postoperative antibiotics; *Group 2*: 2–3 days of postoperative antibiotics; *Group 3*: antibiotic treatment for>3 days; *IQR*: interquartile range.

**Table 2:** Demographic and clinical data of the study groups.

Characteristic	Group 1	Group 2	Group 3	*p*-Value
n (%)	30 (14.3%)	123 (58.9%)	56 (26.8%)	
Sex				0.243
Male	15	44	18	
Female	15	79	38	
Surgical Procedures				0.11
Single Jaw	16	46	17	
Bimaxillary	14	77	39	
Osteotomy Type				
Maxilla *				0.137
SARPE	9	23	5	
LeFort I	19	95	46	
Mandible ^				0.167
IVRO	14	68	35	
SSO	2	14	9	
Genioplasty	5	15	10	0.542
Feeding Tube	9	77	38	0.0008
Catheter	15	76	42	0.065
Preoperative Antibiotics	29	122	54	0.233
Postoperative Steroids	2	8	3	0.999<
Infections	1	3	3	0.642

*Group 1*: one day of postoperative antibiotics; *Group 2*: 2–3 days of postoperative antibiotics; *Group 3*: >3 days of postoperative antibiotics; *IQR*: interquartile range; *SARPE*: surgically assisted rapid palatal/maxillary expansion; *IVRO*: intraoral vertical ramus osteotomy; *SSO*: sagittal split osteotomy. * The rest had undergone mandible-only procedures. ^ The rest had undergone maxilla-only procedures.

**Table 3:** Demographic and clinical data of the surgical-site infected and non-infected groups.

Characteristic	SSI Group	Non-Infected Group	*p*-Value
n (%)	7 (3.3%)	202 (96.7%)	
Sex			0.010
Male	6	71	
Female	1	131	
Surgical Procedures			0.712
Single Jaw	2	77	
Bimaxillary	5	125	
Osteotomy Type			
Maxilla			0.402
None (Mandible Only)	1	11	
SARPE	1	36	
LeFort I	5	155	
Mandible			0.536
None (Maxilla Only)	1	66	
IVRO	5	112	
SSO	1	24	
Genioplasty	1	29	0.999<
Feeding-Tube Use	4	120	0.999<
Catheter Use	5	128	0.999<
Preoperative Antibiotics	7	198	0.999<
Postoperative Steroids	1	12	0.366

*SSI*: surgical site infection; *SARPE*: surgically assisted rapid palatal/maxillary expansion; *IVRO*: intraoral vertical ramus osteotomy; *SSO*: sagittal split osteotomy.

**Table 4:** Characteristic continuous variables of the surgical-site infected and non-infected groups.

Characteristic	SSI Group	Non-Infected Group	*p*-Value
Age (years)			0.632
Median	22	21	
IQR	18–23	18–25.25	
Operation Duration (hours)			0.988
Median	4	4.03	
IQR	2.57–4.95	2.98–4.68	
Hospitalization (days)			0.976
Median	5	6	
IQR	4–8	5–7	
Follow-up (weeks)			0.864
Median	8.43	7.5	
IQR	4.43–29	3.43–28	

*SSI*: surgical-site infection; *IQR*: interquartile range.

## Data Availability

The data that support the findings of this study are available on request from the corresponding author. The data are not publicly available due to ethical and legal restrictions.
